# Targeting Gastrin-Releasing Peptide Suppresses Neuroblastoma Progression via Upregulation of PTEN Signaling

**DOI:** 10.1371/journal.pone.0072570

**Published:** 2013-09-09

**Authors:** Pritha Paul, Jingbo Qiao, Kwang Woon Kim, Carmelle Romain, Sora Lee, Natasha Volny, Bret Mobley, Hernan Correa, Dai H. Chung

**Affiliations:** 1 Department of Pediatric Surgery, Vanderbilt University Medical Center, Nashville, Tennessee, United States of America; 2 Department of Cancer Biology, Vanderbilt University Medical Center, Nashville, Tennessee, United States of America; 3 Department of Pathology, Vanderbilt University Medical Center, Nashville, Tennessee, United States of America; Ospedale Pediatrico Bambino Gesu', Italy

## Abstract

We have previously demonstrated the role of gastrin-releasing peptide (GRP) as an autocrine growth factor for neuroblastoma. Here, we report that GRP silencing regulates cell signaling involved in the invasion-metastasis cascade. Using a doxycycline inducible system, we demonstrate that GRP silencing decreased anchorage-independent growth, inhibited migration and neuroblastoma cell-mediated angiogenesis *in vitro*, and suppressed metastasis *in vivo*. Targeted inhibition of GRP decreased the mRNA levels of oncogenes responsible for neuroblastoma progression. We also identified PTEN/AKT signaling as a key mediator of the tumorigenic properties of GRP in neuroblastoma cells. Interestingly, PTEN overexpression decreased GRP-mediated migration and angiogenesis; a novel role for this, otherwise, understated tumor suppressor in neuroblastoma. Furthermore, activation of AKT (pAKT) positively correlated with neuroblastoma progression in an *in vivo* tumor-metastasis model. PTEN expression was slightly decreased in metastatic lesions. A similar phenomenon was observed in human neuroblastoma sections, where, early-stage localized tumors had a higher PTEN expression relative to pAKT; however, an inverse expression pattern was observed in liver lesions. Taken together, our results argue for a dual purpose of targeting GRP in neuroblastoma –1) decreasing expression of critical oncogenes involved in tumor progression, and 2) enhancing activation of tumor suppressor genes to treat aggressive, advanced-stage disease.

## Introduction

Neuroblastoma is the most common extracranial solid malignancy of childhood with a significant mortality for all tumor stages at 50% [1], [2]. Being of neuroendocrine origin, neuroblastoma secretes various peptides that are tumorigenic and promote characteristic tumor behavior [3]. Among them, gastrin-releasing peptide (GRP), the mammalian equivalent of bombesin (BBS), has been shown to play a key role in the mitogenic potential of neuroblastoma cells [4]. We have reported that GRP stimulates neuroblastoma cell proliferation [4] and promotes PI3K/AKT-mediated cell survival [5]. We also showed that BBS/GRP increases angiogenesis and primary neuroblastoma growth *in vivo* [6]. While we have demonstrated the importance of GRP in the establishment of neuroblastoma at its primary site, its role in tumor progression and metastasis remains to be fully elucidated.

Metastasis – the spread of cancer cells from the primary tumor to distant sites – is typically a hallmark of a more aggressive and chemoresistant phenotype for various cancers, and neuroblastoma is no exception. The process of metastasis requires tumor cell proliferation, angiogenesis and invasion into the local lymphatic and capillary network. Further, metastatic cells must detach and embolize into the systemic circulation, extravasate and arrest into distant organs and, finally, “seed” and survive at distant sites [7]. A key aspect in the transition from primary tumor growth to invasion and metastasis is acquisition of anchorage-independence [8]. Hence, tumor cells must acquire resistance to anoikis, a form of apoptosis induced in cells that become detached from the extracellular matrix [9]. The ability to evade anoikis early on sets the stage for cancer progression and eventual metastasis to other organs.

The patients >18 months of age presenting with metastatic neuroblastoma at diagnosis remain difficult to treat and cure [10]. Therefore, understanding the process of dissemination and invasion-metastasis cascade is critical to developing targeted therapeutic strategies that could prevent tumor progression. GRP is known to increase invasiveness of prostate cells through enhanced motility [11]. However, whether GRP is involved in promoting metastasis and by what mechanism this may occur in neuroblastoma has not been answered. Given its crucial function in primary neoplasm growth, we sought to determine the role of GRP in neuroblastoma invasion and metastasis.

In this report, we show that silencing of GRP signaling has a negative effect on the invasion-metastasis cascade in neuroblastoma cells. Our results demonstrate that GRP silencing leads to upregulation of phosphatase and tensin homologue (PTEN), a negative regulator of the PI3K/AKT pathway, with a simultaneous decrease in the expression of phosphorylated AKT (pAKT) and mTOR (pmTOR). We also identified new downstream targets of GRP in neuroblastoma that are known to be responsible for tumor progression. Furthermore, *in vitro* migration of cancer cells and tubule formation by human umbilical vein endothelial cells (HUVECs) demonstrate that PTEN overexpression decreased GRP-mediated motility and angiogenesis in neuroblastoma potentially through decreased activation of AKT and/or FAK. Importantly, using a tissue microarray we observed an inverse correlation between PTEN expression and AKT activation in metastatic lesions from liver when compared to localized disease. Finally, we demonstrate that GRP silencing reduced primary tumor growth and inhibited liver metastasis in our *in vivo* tumor-metastasis model. Taken together, our findings illustrate the significance of GRP in promoting tumor migration, invasion and metastasis and make it a promising target in preventing a more aggressive, malignant neuroblastoma phenotype.

## Materials and Methods

### Materials

Antibodies against pAKT (S473), AKT, PTEN, pmTOR, mTOR, TWIST, MYCN and cell lysis buffer were obtained from Cell Signaling Technology (Beverly, MA). Antibodies against focal adhesion kinase (FAK) and phosphorylated FAK (pFAK) were from BD Biosciences (Franklin Lakes, NJ). Horseradish peroxidase (HRP)-conjugated secondary antibodies against mouse and rabbit IgG were obtained from Santa Cruz Biotechnology, Inc. (Santa Cruz, CA). Enhanced chemiluminescence (ECL) HRP substrates were purchased from Millipore (Immobilon Western) and Perkin Elmer (Western Lightning). Primers for *MYCN*, *FAK* and *TWIST* were designed using Primer-BLAST and ordered from Sigma-Aldrich (St. Louis, MO). Doxycycline was purchased from Sigma-Aldrich. GRP was purchased from Bachem (Torrance, CA).

### Cell culture, plasmids and transfection

Human neuroblastoma cell lines, BE (2)-C (*MYCN*-amplified) and SH-SY5Y (*MYCN* non-amplified), was purchased from American Type Culture Collection (Manassas, VA). Cells were maintained in RPMI 1640 media with L-glutamine (CellGro Mediatech, Inc. Herndon, VA) supplemented with 10% FBS. Cells were maintained at 37°C in a humidified atmosphere of 95% air and 5% CO_2_. For transfection, cells were plated in 6-well plates and transfected with plasmids (total of 4 μg). Vectors pBP_2_ and pBP_2_-HA-PTEN were gifts from Dr. Webster Cavenee (Univ. of California, San Diego, CA).

### Inducible knockdown system

For knockdown of our target gene, human GRP, we used BLOCK-iT Inducible H1 Lentiviral RNAi System (Life Technologies, Invitrogen, Grand Island, NY). The sequence targeting GRP (NM_002091) is underlined in the shRNA (shGRP) sequence: 5′-CACCAGCAATCAGCAGCCTTCGTGGGACGAATCCCACGAAGG CTGCTGATTGC-3′; the nonspecific control shCON is: 5′-CACCGGGCGCGCTTTGT AGGATTCGCCG AAGCGAATCCTACAAAGCGCGCC-3′. shRNA sequences were cloned into the BLOCK-iT Inducible H1 RNAi Entry Vector (pENTR^TM^/H1/TO), and then shRNA was inserted into Lentiviral vector pLenti4/BLOCK-iT-DEST by LR recombination between pENTR^TM^/H1/TO entry and pLenti4/BLOCK-iT expression constructs. Inducible shRNA expression cells were established by transfecting cells with both pLenti6/TR and pLenti4/BLOCK-iT-DEST, or by introducing the vectors with the lentiviral-mediated delivery system. Production of lentivirus was performed in 293FT cells. Stable cell lines BE (2)-C/Tet/shCON, BE (2)-C/Tet/shGRP, SH-SY5Y/Tet/shCON and SH-SY5Y/Tet/shGRP were established by selecting with Blasticidin at 8 μg/ml and Zeocin at 50 μg/ml post lentiviral transductions.

### Immunoblotting

Whole-cell lysates were prepared using cell lysis buffer with 1 mM PMSF and incubated on ice for 30–60 min. Total protein (50 μg) whole-cell lysates were prepared and separated using 4–12% Bis–Tris gels and electrophoretically transferred to polyvinylidene diﬂuoride membranes (BioRad Laboratories, Hercules, CA). Nonspecific binding sites were blocked with 5% milk in TBST (120 mM Tris–HCl, pH 7.4, 150 mM NaCl, and 0.05% Tween 20) for 1 h at room temperature (RT). Target proteins were detected by using rabbit or mouse anti-human antibodies (1∶500–2000 dilution) overnight at 4°C. The membranes were washed three times and incubated with secondary antibodies (1∶10,000 dilution) conjugated with HRP. Immune complexes were visualized using ECL system. Equal loading and transfer were confirmed with β-actin antibody. Data are representative of three independent experiments.

### Tissue microarray construction

For preparation of the neuroblastoma tissue microarray, the surgical pathology specimen database at Vanderbilt Medical Center was searched for neuroblastoma diagnosis from 1992 to 2011 (Vanderbilt IRB protocol #111723). A Beecher Instruments Manual Tissue Arrayer was used to prepare tissue cores from selected regions of archival tissue blocks. Four 1 mm cores were prepared for each tumor case. In general, tissue biopsies were obtained from the adrenal medulla or paraspinal mass for Stage 1–3 patients without metastasis, and from the lung, lymph node or liver for Stage 4 patients with metastasis.

### Immunohistochemistry

Tissues were fixed in formalin for 3 days and embedded in paraffin wax. Paraffin-embedded sections (5 μm) were deparaffinized in three xylene washes followed by a graded alcohol series, antigen retrieval performed with 10 mM sodium citrate buffer, and then blocked with blocking solution for 1 h at RT. Sections were incubated with primary antibodies (PTEN or pAKT) overnight at 4°C. They were washed with phosphate buffered solution (PBS) and incubated with secondary antibodies for 30 min at RT. Sections were developed with DAB reagent. Sections were counterstained with hematoxylin, dehydrated with ethanol and xylene. Coverslips were mounted and slides observed by light microscopy. Blinded scoring (0–3) was performed by a pediatric pathologist with expertise in neuroblastoma (H.C.).

### Soft agar colony formation assay

Cells were trypsinized, resuspended in RPMI 1640 media containing 0.4% agarose and 10% FBS and overlaid onto a bottom layer of solidified 0.8% agarose in RPMI media 1640 containing 5% FBS, at concentrations of 3×10^3^ cells per well in 6-well plates, and incubated for 3 weeks. Colonies were stained with 0.05% crystal violet, photographed, and quantified.

### Migration assay

For transwell migration assay, polycarbonate transwell filters (8 μm; Corning Inc., Corning, NY) were coated on the lower side with 5 μg/ml collagen type I (BD Biosciences) overnight and then blocked with 2.5% BSA in PBS for 1 h. 1×10^5^ cells in 500 μl of serum-free media were added to the transwell and allowed to migrate for 4 h at 37°C under tissue culture conditions. Media with 1% FBS or 100 nM GRP was added to the lower chamber. Cells that failed to migrate through the filter after incubation were scraped out using a sterile cotton swab. Cells that migrated to the bottom surface of the filter were fixed with 4% paraformaldehyde, stained with DAPI, and counted. Each substrate was repeated in duplicate wells, and within each well counting was done in five randomly selected microscopic fields (200X magnification).

### *In vitro* tubule formation assay

HUVECs grown to ∼70% confluence were trypsinized, counted, and seeded with various conditioned media at 48,000 cells per well in 24-well plates coated with 300 μl of Matrigel (BD Biosciences) and treated with cell culture supernatant from transfected BE (2)-C or SH-SY5Y cells. HUVECs were periodically observed by microscope as they differentiated into capillary-like tubule structures. After 6 h, cells were stained with hematoxylin & eosin (H&E) and photographed via microscope. The average number of tubules was calculated from examining three separate microscopic fields (200X) and representative photographs obtained.

### Reverse transcription, semi-quantitative and quantitative real-time polymerase chain reaction (QRT-PCR)

Total RNA was isolated using Trizol and reverse-transcribed to cDNA using High Capacity cDNA reverse transcription kit according to manufacturer's protocol (Applied Biosystems, Foster City, CA). Semi-quantitative PCR was performed using a Peltier Thermal Cycler (PTC-200) using specific 3′and 5′ primers for GRP and final product was visualized on 1% agarose gel using a Gel Doc (Bio-Rad). QRT-PCR was performed using the Bio-Rad Thermocycler CFX96. SsoFAST EvaGreen Supermix, cDNA and specific 3′ and 5′ primers were incubated together using the manufacturer's protocol (Bio-Rad). GAPDH and β-actin were used as internal controls.

### *In vivo* experiments

Male athymic nude mice (4–6 weeks old) were maintained as previously described [6]. All studies were approved by the Institutional Animal Care and Use Committee at Vanderbilt University and were conducted in accordance with NIH guidelines. BE (2)-C cells stably transfected with Tet/shCON or Tet/shGRP was used for animal experiments. Mice were anesthetized with isofluorane/oxygen mixture, and a small left flank incision was made to isolate and exteriorize the spleen. A total of 1×10^6^ cells in 50 μl of HBSS was injected into the splenic capsule using a 27-gauge needle. Abdominal wall was closed with metal wound clips. Mice were randomized to 3 groups: (1) vector-control group BE (2)-C/Tet/shCON (n = 3) was allowed to drink autoclaved water mixed with sucrose (3%) and doxycycline (2 mg/mL), (2) inducible-control group BE (2)-C/Tet/shGRP (n = 4) was given sucrose (3%) alone without doxycycline, and (3) inducible-treatment group BE (2)-C/Tet/shGRP (n = 4) was given sucrose (3%) and doxycycline (2 mg/mL). Mice were weighed weekly and tumor growth was assessed biweekly. At sacrifice, spleens and livers were harvested, weighed and fixed in formalin for analyses.

### Statistical analysis

Scoring index was expressed as means ± SEM for both *in vitro* and *in vivo* experiments; statistical analyses were performed using Student's t-test for *in vitro* and *in vivo* experiments and Kruskal-Wallis one-way analysis of variance by ranks for comparisons between the treatment groups *in vivo*. For immunohistochemistry, quantification was based on blinded scoring by a pediatric pathologist across serial sections from multiple animals or patient samples. Scores were analyzed by Student's t-test for statistical significance. A *p* value of <0.05 was considered significant.

## Results

### Silencing GRP inhibited neuroblastoma tumorigenicity *in vitro*

**Figure 1:**
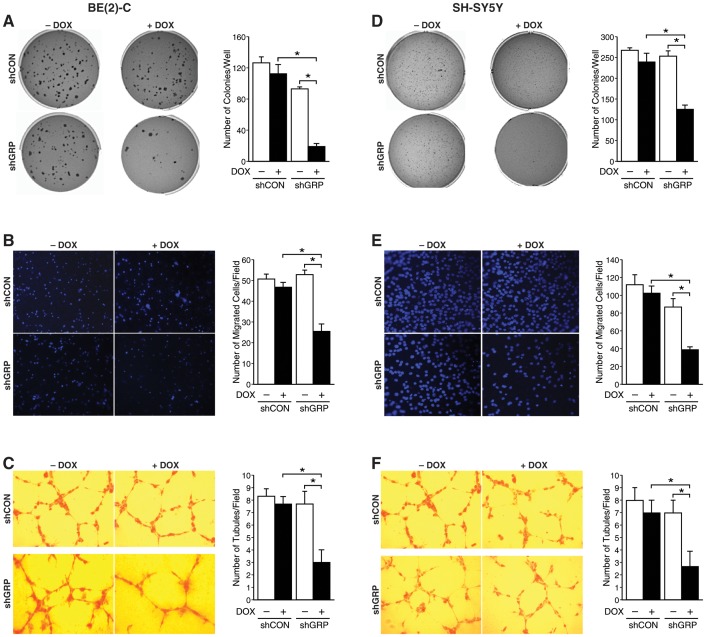
Targeted GRP silencing inhibited neuroblastoma progression. (A, D) BE (2)-C/Tet/shGRP (+DOX) cells and SH-SY5Y/Tet/shGRP (+DOX) cells, respectively, demonstrated a decrease in soft agar colony formation in comparison to Tet/shGRP (-DOX) cells or Tet/shCON (+DOX) cells. (B, E) GRP silencing in BE (2)-C/Tet/shGRP (+DOX) and SH-SY5Y/Tet/shGRP (+DOX) cells, respectively, decreased cell migration in a transwell assay in comparison to controls. (C, F) HUVECs cultured in cell culture supernatant from GRP silenced cells resulted in decreased tubule formation than when grown in supernatant from control cells (mean ± SEM; * = *p*<0.05).

GRP acts as a mitogen for a variety of cancers, including neuroblastoma. We have previously described a function of GRP in inducing neuroblastoma cell cycle progression [5]. Tumor progression requires local migration and invasion, the ability to evade anoikis-induced cell death while disseminating through lymphatic and hematogenous systems to establish tumors at distant sites. Using a doxycycline-inducible system to silence GRP in human neuroblastoma BE (2)-C and SH-SY-5Y cells, we examined the effects of targeting GRP in neuroblastoma progression. Here, we demonstrate that GRP silencing decreased the anchorage-independent growth of neuroblastoma cells, which indicates enhanced anoikis-induced cell death *in vitro*. The number of soft agar colonies after doxycycline treatment-induced GRP silencing was significantly reduced when compared to doxycycline-untreated BE (2)-C/Tet/shGRP cells and doxycycline-treated BE (2)-C/Tet/shCON cells (Fig. 1A). Moreover, doxycycline-induced silencing of GRP also significantly decreased transwell migration of BE (2)-C/Tet/shGRP cells when compared to controls (Fig. 1B). Consistent with decreases in soft agar colony formation and cell migration, HUVECs grown in cell culture supernatant from doxycycline treated BE (2)-C/Tet/shGRP also demonstrated visibly reduced tubule formation in comparison to untreated BE (2)-C/Tet/shGRP cells and BE (2)-C/Tet/shCON cells (Fig. 1C). Similar observations were made with the SH-SY5Y cells transfected with Tet/shCON or Tet/shGRP (Figs. 1D-F). GRP silencing was confirmed by semi-quantitative and real-time PCR (Figure S1). Taken together, these data indicate that targeting GRP affects multiple steps of the invasion-metastasis cascade.

### Silencing GRP downregulated pAKT and oncogenes critical for neuroblastoma progression

**Figure 2:**
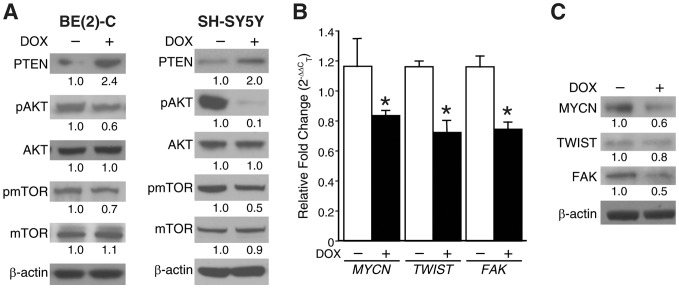
GRP silencing on PTEN/AKT/mTOR signaling and MYCN, TWIST, FAK. (A) BE(2)-C/Tet/shGRP (+DOX) cells and SH-SY5Y/Tet/shGRP (+DOX) cells had an increase in PTEN expression along with correlative decreases in pAKT and pmTOR expression when compared to control cells (without doxycycline; -DOX). (B) Doxycycline-induced GRP silencing in BE (2)-C/Tet/shGRP cells decreased the mRNA levels of *MYCN*, *TWIST* and *FAK* by ∼50–60% as assessed by QRT-PCR (mean ± SEM; * = *p*<0.05 vs. without DOX). (C) Immunoblotting confirmed the decreases in the protein levels of MYCN, TWIST and FAK after GRP silencing (+DOX) in comparison to untreated (-DOX) BE (2)-C/Tet/shGRP cells. β-actin was used as a loading control.

Gastrin-releasing peptide receptor (GRP-R) overexpression downregulated PTEN transcription [12] and GRP treatment induces neuroblastoma cell cycle progression via PI3K/AKT [5]. Much is unknown about the downstream signaling pathways and target genes involved in GRP-mediated neuroblastoma progression. Similar to studies of GRP-R silencing [13], silencing of GRP using doxycycline inducible system increased PTEN expression with a concomitant decrease in pAKT expression and its downstream effector, pmTOR (Fig. 2A). Interestingly, doxycycline-induced GRP silencing in BE (2)-C/Tet/shGRP cells suppressed the transcription of critical oncogenes involved in neuroblastoma progression such as *MYCN*, *TWIST* and *FAK* (Fig. 2B). Correlative to downregulation at the transcriptional level, GRP silencing also decreased protein levels of MYCN, TWIST and FAK (Fig. 2C). Hence, our data indicate that GRP may modulate both the transcription of oncogenes as well as signaling pathways implicated in neuroblastoma progression.

### PTEN overexpression decreased GRP-mediated neuroblastoma progression

**Figure 3:**
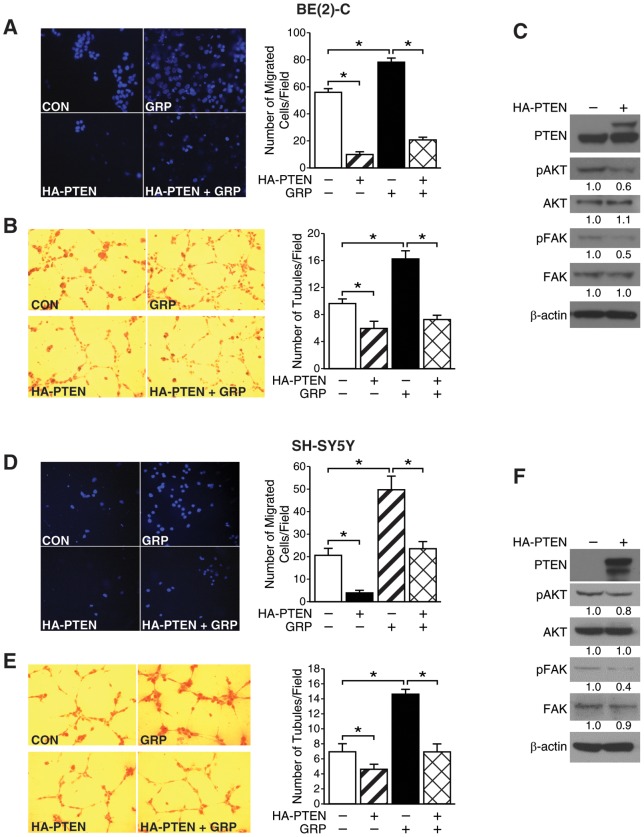
PTEN overexpression inhibited GRP-mediated neuroblastoma progression. (A, D) PTEN overexpression (HA-PTEN) decreased BE (2)-C and SH-SY5Y cell migration in comparison to vector control (CON) as assessed by transwell migration assay (*left panels*). GRP treatment increased BE (2)-C and SH-SY5Y cell migration (*top panels*); this was attenuated by PTEN overexpression (HA-PTEN+GRP) (*right panels*). (B, E) *In vitro* tubule formation by HUVECs grown in the supernatant of PTEN overexpressing BE (2)-C or SH-SY5Y cells (HA-PTEN) was markedly reduced in number in comparison vector control (CON) (*left panels*). GRP-mediated (GRP) increase in HUVEC tubule formation was inhibited when grown in supernatant from PTEN overexpressing BE (2)-C or SH-SY5Y cells (HA-PTEN+GRP) (*right panels*). (C, F) PTEN overexpression in BE (2)-C and SH-SY5Y cells decreased pAKT and pFAK as assessed by immunoblotting. β-actin was used as a loading control (mean ± SEM; * = *p*<0.05).

PTEN negatively regulates cancer cell migration by suppressing the tyrosine phosphorylation of FAK or p130^CAS^ [14]. Therefore, we next examined the role of PTEN overexpression in neuroblastoma cell migration using pBP_2_-HA-PTEN overexpression plasmid or the control vector, pBP_2_. BE (2)-C/HA-PTEN cell migration was significantly reduced compared to BE (2)-C/CON cells when subjected to media containing 1% FBS in the lower chamber (Fig. 3A). In order to ascertain the role of PTEN in inhibiting GRP-mediated migration, we added media containing 1% FBS and 100 nM GRP in the lower chamber. As expected, under reduced serum conditions GRP treatment increased the migratory capacity of BE (2)-C/CON cells in comparison to BE (2)-C/CON cells without GRP (Fig. 3A). Moreover, the number of migrated BE (2)-C/HA-PTEN cells with GRP treatment was significantly lower than BE (2)-C/CON cells with or without GRP (Fig. 3A). Furthermore, PTEN overexpression had a similar inhibitory effect on *in vitro* tubule formation indicating a novel role for PTEN in tumor progression (Fig. 3B). PTEN overexpression could completely block GRP-mediated increase in tubule formation by HUVECs (Fig. 3B). Similar observations were made with SH-SY5Y cells transfected with control vector or PTEN overexpression vector, and subsequently treated with or without GRP (Figs. 3D, E). Immunoblotting demonstrated that PTEN overexpression decreased pAKT and pFAK expression in BE (2)-C and SH-SY5Y cells (Figs. 3C and F, respectively). This set of novel observations implicated that PTEN overexpression inhibited GRP-induced neuroblastoma progression.

### Ratio of PTEN and pAKT expression in human neuroblastoma sections

**Figure 4:**
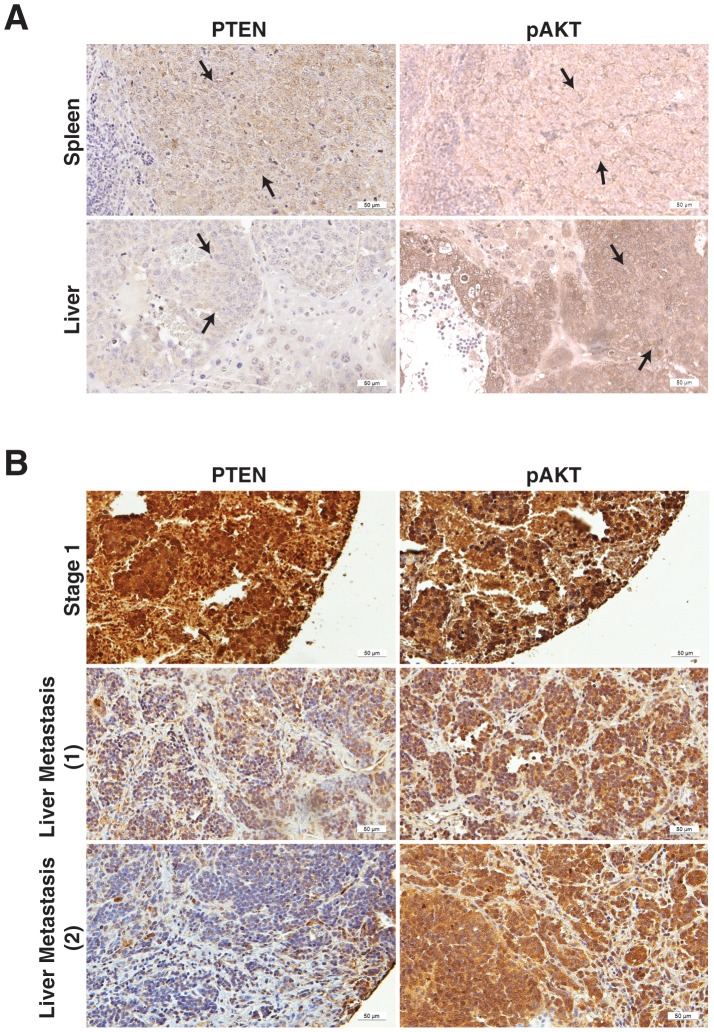
Inverse pattern of PTEN and pAKT expression in metastatic lesions. (A) Immunohistochemistry demonstrated increased pAKT expression in liver lesions (*brown staining; bottom right*) from our *in vivo* spleen-liver metastasis model in comparison to primary splenic tumors, whereas, PTEN expression was slightly decreased in metastatic liver foci when compared to primary splenic tumors (*brown staining; top left*) (black arrows indicate tumors). (B) PTEN expression was lower in liver metastatic sections from human neuroblastoma samples in comparison to sections from primary localized tumors. pAKT expression was comparatively higher in patients with liver metastasis.

Activation of AKT has been correlated with poor prognosis in neuroblastoma patients and indicates disease progression [15]. We have previously identified an inverse correlation between PTEN expression and AKT activation with respect to differentiation in human neuroblastoma samples [12]. To further delve into how PTEN correlates with activation of AKT during neuroblastoma progression, we used an *in vivo* metastasis model established in our laboratory [13] for our study. Human neuroblastoma BE (2)-C cells were injected intrasplenically into mice and liver metastasis occurred in ∼4 weeks. Primary tumors from the spleen as well as liver metastases were harvested, fixed and immunohistochemistry was performed in paraffin-embedded sections. Primary splenic tumors showed a comparatively higher expression of PTEN than the secondary liver lesions (Fig. 4A, *left panel* *s*). Interestingly, there was an increased expression of pAKT in the secondary lesions in the liver in comparison to the primary spleen tumor (Fig. 4A, *right panel* *s*).

To further confirm this inverse correlation of PTEN and pAKT expression in liver metastases from mice study, we next assessed the expression of PTEN and pAKT by immunohistochemistry using a tissue microarray containing human neuroblastoma sections from primary tumor or metastatic lesions at distant organs (Fig. 4B). Neuroblastoma sections obtained from 13 patients at the time of biopsy and/or resection with or without metastasis were chosen for further analyses. Immunohistochemistry analysis of patients with respect to multiple parameters and expression of PTEN/pAKT is summarized in Table 1. We found that there was an inverse correlation of PTEN and pAKT in more advanced-stage disease (i.e., stages 3 or 4), with pAKT expression being relatively higher. Specifically, two thirds of patients (4/6) who had higher PTEN expression were also characterized as having early-stage disease, suggesting that expression of this gene may be a positive prognostic indicator. Interestingly, the other two patients with high PTEN expression were both characterized as having stage 4 diseases with metastases to the lung and lymph nodes. The patients with stage 1 disease, as identified by the international neuroblastoma staging system, had similar expression pattern of PTEN and pAKT (Fig. 4B; *top row*). Similar to our *in vivo* murine metastasis model, expression of pAKT was markedly higher in stage 4 patients with metastasis to the liver compared to PTEN expression (Fig. 4B, *middle and bottom rows*). Taken together, PTEN and pAKT are inversely correlated during neuroblastoma progression.

**Table 1:** PTEN and pAKT expression patterns in human neuroblastoma sections.

Number	Stage	*MYCN* Amp	Risk	Relapse	Metastasis	Status	PTEN	pAKT
1	4	No		Refractory	Lung	Deceased	+++	+
2	4S	No	Low	No		Alive	+++	+
3	4	No			Lymph node	Alive	+++	++
4	4S	Yes		Yes	Liver	Deceased	+	++
5	4	Yes	High	No	Liver	Alive	+	+++
6	4	Yes	High	Yes	Liver	Deceased	+	+++
7	3	No	Intermediate	No		Alive	+	++
8	1	No	Low	No		Alive	+++	+
9	4	No		No	Bone marrow	Deceased	+	+++
10	4	Yes	High	Refractory	Bone marrow	Deceased	–	++
11	3	Yes	High	Yes		Deceased	+	++
12	1	No	Low	No		Alive	+++	++
13	1	No	Low	Yes		Alive	++	+++

–  = absent; +  =  low; ++  =  moderate; +++  =  high.

### Silencing GRP inhibited liver metastasis *in vivo*

**Figure 5:**
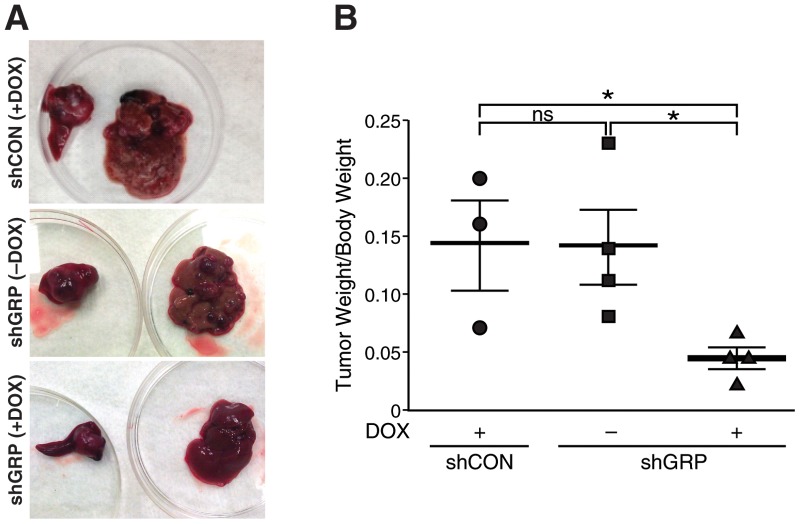
Targeted silencing of GRP inhibited liver metastasis. (A) Large gross tumors were observed in the spleen and liver of mice injected with BE (2)-C/Tet/shCON and subjected to drinking water with doxycycline (2 mg/mL) and 3% sucrose [shCON (+DOX)] or injected with BE (2)-C/Tet/shGRP and subjected to drinking water with 3% sucrose alone [shGRP (-DOX)], whereas, a near complete inhibition of hepatic metastatic lesions was observed in mice subjected to drinking water with doxycycline (2 mg/mL) and 3% sucrose [shGRP (+DOX)]. (B) Liver tumor weights of mice injected with BE (2)-C/Tet/shGRP and receiving doxycycline in drinking water significantly decreased in comparison to control groups (mean ± SEM; * = *p*<0.05).

We next wanted to determine the effects of silencing GRP on neuroblastoma tumor growth and metastasis using our murine metastasis model. Mice intrasplenically injected with BE (2)-C/Tet/shCON (vector-control group) received water containing doxycycline and sucrose. Mice intrasplenically injected with BE (2)-C/Tet/shGRP were further randomized into two groups: (A) Inducible-treatment group receiving doxycycline and sucrose in drinking water, and (B) the inducible-control group receiving sucrose alone. Silencing GRP did not significantly reduce growth of primary tumors in murine spleen in comparison to mice administered with only sucrose in their drinking water (Fig. 5A; *bottom left*). Interestingly, large liver lesions were observed in mice from the vector-control group receiving doxycycline and inducible-control group without doxycycline compared to inducible-treatment group receiving doxycycline treatment, indicating that silencing GRP inhibits establishment of macrometastases in the liver (Fig. 5A, *top right*). Statistical analyses indicated a significant decrease in the liver tumor burden in mice injected with BE (2)-C/Tet/shGRP and receiving doxycycline in drinking water in comparison to the controls (Fig. 5B). These data demonstrate the critical role of GRP in metastasis to secondary sites and a potential use of targeting GRP in treating aggressive, advanced-stage neuroblastomas.

## Discussion

The presence of metastatic disease is a harbinger of poor clinical outcome and, unfortunately, decreased survival. Because of this fact, the importance of determining the mechanisms by which cancer cells undergo hematogenous and/or lymphatic dissemination and become metastatic cannot be understated. We have previously demonstrated an increased expression of GRP and its receptor, GRP-R, in the more aggressive undifferentiated neuroblastoma 13]. In the present study, we identify that key oncogenic properties, such as anchorage-independence, migration and angiogenesis, required for tumor invasion and metastasis, were all negatively affected by GRP silencing.

Due to our prior knowledge that PTEN mRNA and protein expression is negatively impacted by GRP-R overexpression and that GRP treatment stimulates the PI3K/AKT pathway in neuroblastoma cells [5], we sought to determine whether GRP-mediated signaling regulates PTEN expression. Inhibition of GRP led to increased expression of PTEN, a negative regulator of the PI3K/AKT pathway, which is known to activate tumor proliferation and survival signaling [16]. Furthermore, targeted inhibition of GRP suppressed the activation of the AKT/mTOR signaling cascade and transcription of critical oncogenes involved in neuroblastoma progression, specifically *MYCN*, *TWIST* and *FAK*. It is well established that *MYCN* amplification is one of the strongest predictors of advanced disease, tumor progression and poor clinical outcome in patients with neuroblastoma [17]. Interestingly, in conjunction with *MYCN* amplification, the transcription factor TWIST has been shown to prevent the apoptotic response by inhibiting the ARF-p53 pathway in neuroblastoma [18]. FAK, a nonreceptor protein kinase, has a significant role in many cellular pathways including cellular adhesion and migration [19], especially in neuroblastoma [20]. Transcriptional regulation of these well-established oncogenes by GRP highlights its role in promoting metastasis.

Early work on PTEN in neuroblastoma suggested that only a small number of cell lines harbor mutations in this gene that could contribute to oncogenesis and malignant tumor progression [21], [22]. These initial studies seemed to indicate that significance of PTEN was, at best, marginal and that its role as a tumor suppressor may not be critical in neuroblastoma. Previous work from our laboratory had demonstrated that decreased expression of PTEN was noted in more undifferentiated neuroblastomas and overexpression of GRP-R downregulated PTEN expression [12]. This association between PTEN and a more undifferentiated neuroblastoma phenotype suggested that PTEN could potentially regulate molecular pathways associated with invasion and metastasis.

A diverse group of “molecular sensors”, including cell adhesion molecules, integrins and ligands act in concert with one another to regulate anoikis [23]. These cellular molecules initiate signaling cascades that maintain a pro-apoptotic balance when cellular detachment occurs. Consequently, molecular targets, which can suppress aberrant cell signaling pathways that promote resistance to anoikis, have become the focus of many investigations. Interestingly, PTEN plays a critical role in regulating anoikis [24] and overexpression of this gene inhibits cell migration and invasion in many different cell lines [14], [25]. In concert with its inhibitory role in migration and invasion, restoration of the cellular function of PTEN has been shown to induce anoikis in glioma cell lines via suppression of AKT phosphorylation [26]. Independent of the PI3K/AKT pathway, phosphatase activity of PTEN has also been shown to act on FAK and initiate anoikis [27]. In a similar fashion, we demonstrated that PTEN overexpression reduced neuroblastoma cell migration and tumor-mediated angiogenesis in BE (2)-C cells with concomitant suppression of pAKT and pFAK protein expression. Our findings suggest that PTEN has a crucial role in neuroblastoma, specifically directed at inhibiting cellular processes that promote resistance to anoikis and a pro-metastatic phenotype. Most importantly, PTEN overexpression blocked GRP-mediated tumor progression as assessed by *in vitro* functional assays, thereby, demonstrating the critical role of PTEN in reversing the oncogenic roles of GRP in neuroblastoma.

Our results demonstrated that silencing GRP has a negative effect on the development of characteristics necessary for invasion and metastasis. Previous studies ascertaining the efficacy of GRP antagonists in cancer have focused on its mitogenic property with the aid of subcutaneous xenograft models [6], [20]. To our best knowledge, this is the first report of targeted inhibition of GRP with respect to metastatic disease *in vivo*. Using our above mentioned metastasis model, we were able to demonstrate that targeting GRP inhibited tumor metastasis. Lack of complete inhibition of primary tumor growth could be potentially due to a marginal reduction of the proliferative capacity of neuroblastoma cells after GRP silencing [28]. The result of these *in vivo* experiments illustrates the inhibition of several key features in the invasion-metastasis cascade. Taken together, our results are significant because it identifies a rationale for targeted therapy against GRP to modulate signaling pathways that contribute to neuroblastoma metastasis.

Focused efforts are needed to improve the clinical outcomes of children with advanced-stage, aggressive neuroblastomas and create specific therapeutic treatments that block molecular pathways contributing to resistant and metastatic disease. In this study we have identified that GRP silencing can negatively impact neuroblastoma progression in several ways. Functionally, it appears to inhibit critical steps that are required for metastasis including, anchorage-independence, migration, and angiogenesis. Mechanistically, GRP silencing resulted in upregulation of tumor suppressor PTEN with subsequent downregulation of critical oncogenes and proliferation/survival pathways implicated in neuroblastoma progression. Combination therapies using cytotoxic chemotherapeutic agents and GRP antagonists to targeting metastatic disease would be of significance in treating aggressive neuroblastoma in the future.

## Supporting Information

Figure S1 **Doxycycline treatment induced GRP silencing in neuroblastoma cell lines.** (A) Semi-quantitative analysis of *GRP* mRNA levels confirmed silencing after doxycycline treatment for 48 h in BE (2)-C/Tet/shGRP and SH-SY-5Y/Tet/shGRP. (B) Real-time PCR demonstrated significant silencing of *GRP* mRNA levels after doxycycline treatment for 48 h in BE (2)-C/Tet/shGRP and SH-SY5Y/Tet/shGRP (mean ± SEM; * = *p*<0.05 vs. without doxycycline).
(EPS)
